# Characterization and Engineered U1 snRNA Rescue of Splicing Variants in a Turkish Neurodevelopmental Disease Cohort

**DOI:** 10.1155/2024/7760556

**Published:** 2024-05-28

**Authors:** Ece Sönmezler, Cristiana Stuani, Semra Hız Kurul, Serdal Güngör, Emanuele Buratti, Yavuz Oktay

**Affiliations:** ^1^ Izmir Biomedicine and Genome Center Dokuz Eylul University Health Campus 35340 Izmir, Türkiye; ^2^ Izmir International Biomedicine and Genome Institute Dokuz Eylul University 35340 Izmir, Türkiye; ^3^ International Centre for Genetic Engineering and Biotechnology (ICGEB) 34149 Trieste, Italy; ^4^ Department of Paediatric Neurology School of Medicine Dokuz Eylul University 35340 Izmir, Türkiye; ^5^ Department of Paediatric Neurology School of Medicine Inonu University Malatya 44210, Türkiye; ^6^ Department of Medical Biology School of Medicine Dokuz Eylul University Izmir 35340, Türkiye

## Abstract

Although they are rare in the population, rare neurodevelopmental disorders (RNDDs) constitute a significant portion of all rare diseases. While advancements in sequencing technologies led to improvements in diagnosing and managing rare neurodevelopmental diseases, accurate pathogenicity classification of the identified variants is still challenging. Sequence variants altering pre-mRNA splicing make up a significant part of pathogenic variants. Despite advances in the *in silico* prediction tools, noncanonical splice site variants are one of the groups of variants that pose a challenge in their clinical interpretation. In this study, we analyzed the effects of seven splicing variants we had previously proposed as disease-causing and demonstrated that all but one of the seven variants had a strong or moderate effect on splicing, as assessed by a minigene assay. Next, applying U1 snRNAs engineered for different splicing variants in the corresponding genes and expressed with minigene plasmids in HeLa cells provided a partial correction in four of the studied genes to varying degrees. Findings from our study highlight the importance of in vitro minigene-based assays for the reclassification of putative splice-altering variants of uncertain significance and the therapeutic potential of modified U1 snRNAs in RNDDs.

## 1. Introduction

Rare neurodevelopmental disorders (RNDDs) are known to account for the vast majority of all rare diseases, primarily affecting brain development and function [1]. RNDDs represent a clinically and genetically heterogeneous group of disorders that pose a major challenge for diagnosis and treatment. Around 80% of all rare diseases have a genetic origin, and they are mostly pediatric onset. More than 90% of rare diseases still lack approved therapy [2, 3].

The diagnostic approach to rare diseases has improved with next-generation sequencing (NGS) technologies. The most common NGS technologies, whole exome sequencing (WES) and whole genome sequencing (WGS), have provided a better understanding of genetic heterogeneity in rare diseases and allowed the investigation of new disease genes and variants. Identification of the molecular basis of rare diseases and providing accurate diagnosis can then lead to improvement in treatment and the discovery of potential therapeutic options [4, 5].

However, the study of RNDDs is still difficult because of our inability to determine the clinical significance of all variants from the data generated by NGS. Nearly half of all disease-causing variants are splice-affecting variants, which are also an important contributor to the development of rare diseases [6]. However, because of the very high complexity of the pre-mRNA splicing process, most of the splice-affecting variants outside the canonical splice sites are classified as variants of uncertain significance (VUSs) according to the American College of Medical Genetics and Genomics (ACMG) guidelines. Nonetheless, it is now clear that the contribution of the near-splice region, deep intronic, and exonic splice-altering variants to disease onset and duration is also at a considerable level. In many cases, these variants can disrupt splicing by creating cryptic splice sites or altering splice regulatory elements (SREs) such as intronic/exonic splice enhancers (ISE/ESE) and intronic/exonic splice silencers (ISS/ESS). Abnormal splicing events mainly include exon skipping which is the most common type of mis-splicing, partial exon skipping, and partial intron retention. For this reason, elucidating these variants' pathogenicity is crucial for their correct interpretation and accurate diagnosis.

While computational tools for splicing defect prediction provide evidence for assessing the pathogenicity of splicing variants, their efficiency is far from completely satisfactory. Therefore, RNA-based analyses are needed to understand better the splicing pattern and correct variant classification [7–9].

In vitro minigene splicing assay, a feasible approach when there is limited accessibility of patient sample, relies on the plasmid-based technique consisting of a genomic segment including the exon to be studied and its short intronic flanking segments [10]. Correspondingly, minigene splicing assay has been recently used in the functional characterization of putative splicing variants in several neurodevelopmental diseases [11–14].

Therapeutic modulation of the aberrant splicing is possible with the established approaches such as the use of engineered U1 small nuclear RNA (U1 snRNA) that is involved in the early steps of spliceosome assembly and splice-switching oligonucleotides (SSO) that regulate splicing by blocking the interactions between the pre-mRNA and splicing factors [15, 16]. Engineered U1 snRNA therapies include modified U1 snRNAs and the second generation of modified U1 snRNAs called exon-specific U1 snRNAs (ExSpeU1s). Modified U1 snRNAs, with compensatory changes at the 5′ tail, increase the recognition of authentic 5′ splice sites (5′ss) impaired by nucleotide variations. The new-generation modified U1 snRNAs, ExSpeU1s, act by targeting the intronic region downstream of the 5′ss of a defective exon to recruit splicing factors for spliceosome assembly. Differently from modified U1 snRNAs, which correct splicing defects caused by 5′ss changes, ExSpeU1s can restore defective splicing caused by changes either at the 5′ss and 3′ss or in exonic regions [17]. To this date, several studies have shown the therapeutic efficacy of the engineered U1 snRNAs both in vitro and in vivo for different neurological conditions [18–21].

In our recent cohort study of 246 pediatric patients from consanguineous Turkish families, WES provided an 86% diagnostic rate [22]. Among the identified variants, ~10% were splice site or near splice site variants.

In the present study, we have now analyzed seven putative splice-affecting variants classified as mostly likely pathogenic or VUS. The first one is a single nucleotide variant ((NM_000124.4): c.1992+3A>G) in *ERCC6* (ERCC excision repair 6, chromatin remodeling factor) gene coding for a protein involved in the transcription-coupled nucleotide excision repair and mostly associated with rare Cockayne syndrome type B (CSB) (OMIM: 133540) [23]. The second one is a missense variant ((NM_175732.3): c.255G>C (p.Gln85His)) in *PTPMT1* (protein tyrosine phosphatase mitochondrial 1) gene encoding phosphoinositide lipid phosphatase that has been shown to regulate, cardiolipin biosynthesis, mitochondrial membrane integrity, and ATP production. Intriguingly, the global and conditional knockout of the gene in mouse models caused embryonic lethality and developmental abnormalities, respectively [24, 25]. The third one is another single nucleotide variant ((NM_001923.5): c.2566+4A>G) in the *DDB1* (damage-specific DNA-binding protein 1) gene that is also coding for a protein involved in the nucleotide excision repair of the UV-damaged DNA and associated with White-Kernohan syndrome (WHIKERS) (OMIM: 619426) [26]. The next variant ((NM_001101426.4): c.1026+6T>A) is in the *CRPPA* (CDP-L-ribitol pyrophosphorylase A) gene (previous gene symbol is *ISPD*) coding for a protein involved in the glycosylation of the *α*-dystroglycan which is an extracellular matrix protein (ECM) responsible for the maintenance of ECM-cell interaction mainly in the muscles, nerves, and brain. Muscular dystrophy-dystroglycanopathy (congenital with brain and eye anomalies), type A, 7 (OMIM: 614643), and muscular dystrophy-dystroglycanopathy (limb-girdle), type C, 7 (OMIM: 616052) are diseases linked to the *CRPPA* gene [27, 28]. Another variant studied in the present study is an intronic insertion variant ((NM_001316320.2): c.1116+2_1116+3insTT) in *PLOD1* (procollagen-lysine, 2-oxoglutarate 5-dioxygenase 1) gene that provides instructions to make a lysyl hydroxylase enzyme enabling the conversion of lysine to hydroxylysine which is found in collagen-like peptides. Accordingly, the Ehlers-Danlos syndrome, kyphoscoliotic type, 1 (OMIM: 225400), is a disease associated with the *PLOD1* gene [29]. The sixth variant is a single nucleotide variant ((NM_014149.4): c.1395+1G>A) in *WDR91* (WD repeat domain 91) gene coding for an endosomal protein which is a regulator of phosphatidylinositol 3-kinase (PI3K) complex via Rab7 interaction to regulate early to late endosome conversion. Studies showed that the conditional knockout of *WDR91* in neurons in mice caused the accumulation of intermediate endosomes and developmental abnormalities including impaired brain development [30, 31]. Finally, the last variant ((NM_001040142.2): c.1177-2A>C) is a single nucleotide variant in the *SCN2A* (sodium voltage-gated channel alpha subunit 2) gene coding for a voltage-gated sodium channel involved in the muscles and neurons to regulate the cellular excitability. The *SCN2A* gene is commonly associated with developmental and epileptic encephalopathy 11 (OMIM: 613721); episodic ataxia, type 9 (OMIM: 618924); and seizures, benign familial infantile, 3 (OMIM: 607745) [32].

Most of these genes are previously reported known disease genes. However, there are also novel candidate disease genes (*PTPMT1* and *WDR91*) that were first reported in our cohort study [22].

Using the minigene splicing assay, we have functionally evaluated their effects on the splicing process of the associated genes and then tested the effect of modified U1 snRNAs to correct splicing defects in these genes.

## 2. Materials and Methods

### 2.1. Patients and *In Silico* Predictions

The study included seven pediatric Turkish patients with neurodevelopmental conditions (Table 1). After the analysis of the data from WES by applying standard filtering criteria including minor allele frequency (MAF) < 1% and high to moderate variant effect prediction (VEP) as previously described [22], *in silico* predictions for the effects of the identified splicing variants were performed by using Human Splicing Finder (HSF) 3.1 (https://www.genomnis.com/the-system-1) and varSEAK (https://varseak.bio/) online tools [33, 34]. The top seven variants with a high splicing effect score were selected for minigene-based splicing analysis and U1 snRNA-mediated correction.

### 2.2. Minigene Constructs

Wild-type (WT) and mutant minigene constructs for *ERCC6* Exon 9 (c.1992+3A>G), *DDB1* Exon 20 (c.2566+4A>G), *CRPPA* Exon 7 (c.1026+6T>A), *PLOD1* Exon 10 (c.1116+2_1116+3insTT), *WDR91* Exon 9 (c.1395+1G>A), and *SCN2A* Exon 10 (c.1177-2A>C) were generated by cloning the exon of interest along with 100 bp of flanking 5′ and 3′ intronic sequences for each gene into pTB minigene plasmid, which had been digested with NdeI. Alpha-globin-fibronectin EDB (pTB) hybrid minigene construct has been previously described [35]. These WT and mutated minigenes for each gene were purchased from GenScript (Piscataway, New Jersey, USA).

For the *PTPMT1* Exon 2 (c.255G>C) mutant and WT minigene constructs, a genomic region including exon 2 and the flanking intronic regions (42 bp from intron 1 and 245 bp from intron 2) was amplified from the patient's and healthy control's genomic DNA, respectively. NdeI restriction site containing primer sequences used for the PCR amplification is given in Supplementary Table S1. Subsequently, PCR products were digested with NdeI enzyme, purified using NucleoSpin® Gel and PCR Clean-up kit (Macherey-Nagel™, Düren, Germany), and cloned into the NdeI digested pTB minigene vector. The orientation and the sequence of the mutant and WT fragments were confirmed through Sanger sequencing.

### 2.3. U1 snRNA Expression Constructs

A WT U1 snRNA expression was performed by using the standard pSP64 poly(A) vector. Modified U1 snRNAs for each variant were generated using QuikChange Site-Directed Mutagenesis Kit (Agilent Technologies, Santa Clara, CA, USA) following the manufacturer's instructions, and desired nucleotide changes were confirmed through Sanger sequencing. For each modified U1 snRNA construct, primer sequences used for the PCR reaction are given in Supplementary Table S1.

### 2.4. Cell Culture and Transfection Experiments

HeLa cells (supplied by ATCC®, Manassas, VA, USA), as well as patient and control dermal fibroblasts, were cultured with Dulbecco's modified Eagle medium, DMEM (Gibco™, Thermo Fisher Scientific, Inc., Waltham, MA, USA) supplemented with 10% fetal bovine serum and a 1% antibiotic-antimycotic solution (Sigma-Aldrich®, St. Louis, MO, USA), under conditions of 37°C with 5% CO_2_.

The day before transfection, HeLa cells were seeded in 6-well plates to become 80-90% confluent on the day of transfection. Cells were transiently transfected with 2 *μ*g of each WT or mutant minigene and cotransfected with 0.5 *μ*g of each mutant minigene and 1.5 *μ*g of each WT or modified U1 snRNA plasmid by using the Effectene® Transfection Reagent (Qiagen GmbH, Hilden, Germany) according to the manufacturer's instructions.

### 2.5. Reverse Transcription-Polymerase Chain Reaction (RT-PCR) Analysis

Total RNA isolation from HeLa cells incubated for 48 hours post-transfection and patient and control dermal fibroblasts was performed using RNeasy® Mini Kit (Qiagen GmbH, Hilden, Germany) according to the manufacturer's instructions, and ~1 *μ*g of each RNA sample was converted to cDNA using M-MLV reverse transcriptase enzyme (Invitrogen, Waltham, Massachusetts, USA) and random hexamer primers. While PCR amplification of the fragments including the exon of interest for each case in transiently transfected HeLa cells was performed using standard primers used for the pTB minigene plasmid, amplification of the fragments including the *PTPMT1* exon 2 in patient and control dermal fibroblasts was performed using *PTPMT1* specific primers (Supplementary Table S1). RT-PCR products were visualized on 1.2% agarose gel, and some of them were sequenced to understand their identity.

### 2.6. Western Blotting

Protein extraction from patient and control dermal fibroblasts was performed using the NucleoSpin® TriPrep Kit (Macherey-Nagel™, Düren, Germany) according to the manufacturer's instructions, and 20 *μ*g of protein for each sample was resolved in an SDS-PAGE gel. Following electrophoresis, the proteins were transferred onto a PVDF membrane, which was then blocked using 5% nonfat skim milk in TBST solution. Antibodies targeting PTPMT1 (diluted 1 : 500, Santa Cruz Biotechnology, Inc., sc-390901) and *β*-actin (diluted 1 : 3000, Abcam, Ab8227) were utilized for immunoblotting analysis.

### 2.7. Statistical Analysis

Densitometric analyses of each band on agarose gel images were performed using the ImageJ software (NIH, Bethesda, MD, USA), with subsequent normalization of band intensities relative to the *GAPDH* levels. Statistical analyses were performed using GraphPad Prism 10.2.0 (GraphPad Software, Inc., San Diego, CA), employing two-way analysis of variance (ANOVA) and Bonferroni's post hoc test for comparison among multiple groups. The semiquantitative RT-PCR densitometric data are represented as ± standard error of the mean (SEM) (*n* = 3). Statistical significance was indicated by asterisks (^∗^*P* < 0.05; ^∗∗^*P* < 0.01; ^∗∗∗^*P* < 0.001; ^∗∗∗∗^*P* < 0.0001).

## 3. Results

### 3.1. Patients and Selection of Splicing Variants

For all splicing variants recently identified in our cohort, seven variants with a high splicing effect score and rescue potential with a modified U1 snRNA were chosen for this study. Detailed information on variants and major clinical findings of patients is given in Table 1. All seven variants were homozygous, and they segregate with the disease within each family.

Previously reported homozygous splice site variants c.1992+3A>G (NM_000124.4) in *ERCC6* [36] and c.1116+2_1116+3insTT (NM_001316320.2) in *PLOD1* [37] were predicted to disrupt authentic 5′ss (HSF 3.1 delta score -12.26%, MaxEnt delta score -44.08%) and create new 3′ss (HSF 3.1 delta score -12.26%) with *in silico* tools (HSF and varSEAK), respectively.

*In silico* prediction tools also predicted disruption of the authentic 5′ss for the novel homozygous variants c.255G>C (NM_175732.3) in *PTPMT1* (HSF 3.1 delta score -11.79%, MaxEnt delta score -42.6%), c.2566+4A>G (NM_001923.5) in *DDB1* (HSF 3.1 delta score -10.94%), c.1026+6T>A (NM_001101426.4) in *CRPPA* (MaxEnt delta score -35.61%), and c.1395+1G>A (NM_014149.4) in *WDR91* (HSF 3.1 delta score -29.69%, MaxEnt delta score -80.99%) and disruption of the authentic 3′ss for the novel homozygous variant c.1177-2A>C (NM_001040142.2) in *SCN2A* (HSF 3.1 delta score -31.78%, MaxEnt delta score -79.45%).

### 3.2. The Minigene Assay Revealed Mis-splicing for All Seven Variants

The minigene assay showed altered pre-mRNA splicing to varying degrees for the novel variants and confirmed mis-splicing for the reported ones.

**Figure 1:**
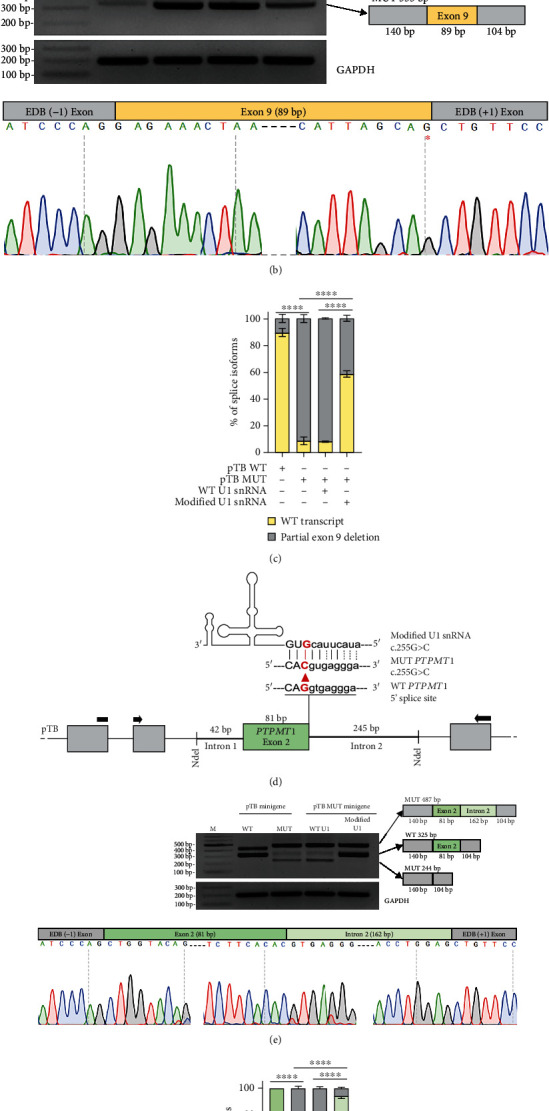
Mis-splicing caused by *ERCC6* c.1992+3A>G and *PTPMT1* c.255G>C variants can be partially rescued by modified U1 snRNAs. (a, d) Schematic representations of the location of variants on pTB minigene clones and the base pairing between the modified U1 snRNAs and the 5′ss of mutant exons. pTB exons and the exon of interests are represented by grey and colored boxes, respectively. Intronic sequences of pTB and flanking intronic regions of the exon of interest are represented by thin and thick lines, respectively. Black arrows stand for forward and reverse primers for RT-PCR amplification. (b, e) RT-PCR analysis of HeLa cells transfected with WT/mutant pTB minigenes and WT/modified U1 snRNAs. RT-PCR products were sequenced and schematically illustrated. The asterisk indicates the last nucleotide (*ERCC6* (NM_000124.4): c.1910) of the partially excluded *ERCC6* exon 9. (c, f) The histogram represents the densitometric analysis of the splicing products normalized to GAPDH (∗∗∗∗ represents *P* < 0.0001). Data are presented as the mean ± SEM (*n* = 3). M: marker; WT: wild type; MUT: mutant.

The c.1992+3A>G variant in *ERCC6* intron 9 was previously shown to cause the use of an alternative 5′ss within exon 9 in patient dermal fibroblasts from another Turkish family. Although the effect of the variant on pre-mRNA splicing was shown in the published study, before proceeding with the U1 snRNA rescue approach, the effect of the c.1992+3A>G variant was checked in HeLa cells by transfecting them with WT and MUT *ERCC6* pTB minigenes covering exon 9 and flanking intronic sequences. Transfection with the WT *ERCC6* minigene resulted in a correctly spliced 415 bp product while transfection with MUT *ERCC6* minigene resulted in a 333 bp product which represents the partial (82 bp) exclusion of the 3′ end of exon 9 due to the activation of an exonic cryptic 5′ss (GT dinucleotide at the position after *ERCC6* (NM_000124.4): c.1910) (Figure 1(b)). Partial exclusion of exon 9 was reported to result in an out-of-frame premature stop codon (p.Arg637Serfs∗34) [36]. While the authentic 5′ss score moderately decreases (HSF 3.1 score from 90.13 to 87.29), the new cryptic 5′ss (GT dinucleotide at the position +3 and +4) created by the c.1992+3A>G variant still has a weaker splicing score (HSF 3.1: 66.49) compared to the score of the disrupted authentic 5′ss site. Nonetheless, partial exon exclusion is thus caused by the activation of an exonic cryptic 5′ss that has a higher splicing score (HSF 3.1: 90.13) at the given position.

Analysis of *PTPMT1* cDNA from primary dermal fibroblast cultures of the patient FIZM011 revealed that the c.255G>C variant at the end of exon 2 leads to aberrant splicing by retaining 162 bp from intron 2. Sanger sequencing revealed that the two predominant products resulting from RT-PCR on patient cDNA include one transcript with 162 bp intron 2 inclusion and exon 3 skipping (486 bp) and another transcript with 162 bp intron 2 inclusion and inclusion of all four exons (678 bp). The 162 bp intronic inclusion introduced an in-frame premature termination codon at the twentieth amino acid position after the end of exon 2 and led to the complete absence of the PTPMT1 protein due to nonsense-mediated mRNA decay (Supplementary Figure S1).

Before proceeding with the rescue approach, to analyze the effect of the c.255G>C variant in the exon 2 of the *PTPMT1* gene in HeLa cells, cells were transfected with WT and MUT *PTPMT1* pTB minigenes covering exon 2 and flanking intronic sequences. Transfection with WT *PTPMT1* minigene resulted in two products: a correctly spliced 325 bp product and an aberrant 427 bp product including 60 bp from the 3′ end of the pTB-resident EDB-1 intron and 42 bp from the *PTPMT1* intron 1 and correctly spliced 325 bp fragment. Transfection of HeLa cells with MUT *PTPMT1* minigene resulted in three products: 487 bp, 325 bp, and 244 bp. Sanger sequencing showed that the favored 487 bp product consisted of the partial retention (162 bp) of intron 2. The 325 bp product represented the correctly spliced transcript and the 244 bp product corresponded to the total exclusion of exon 2 (Figure 1(e)).

**Figure 2:**
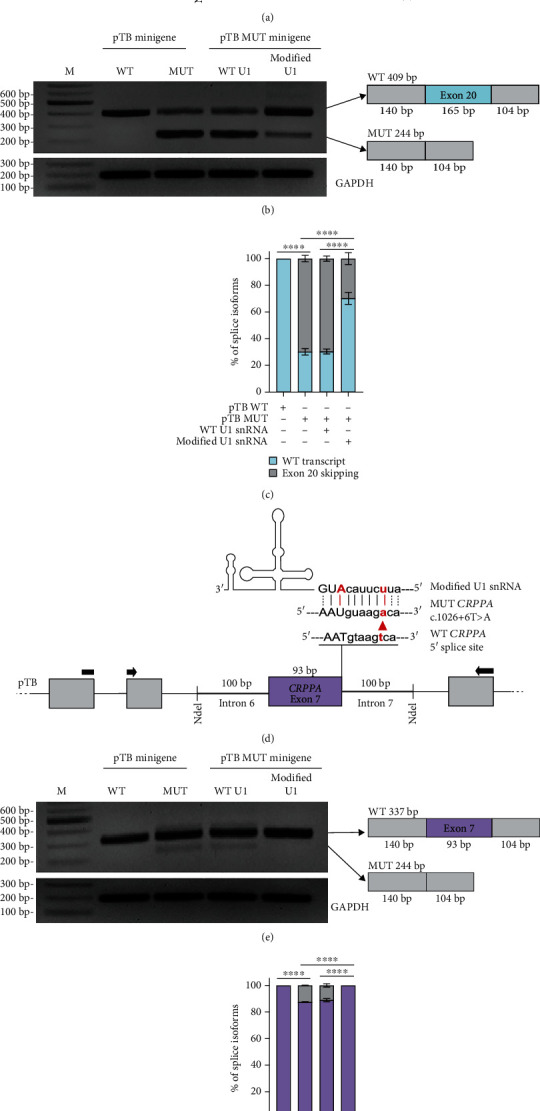
Mis-splicing caused by *DDB1* c.2566+4A>G and *CRPPA* c.1026+6T>A variants can be partially rescued by modified U1 snRNAs. (a, d) Schematic representations of the location of variants on pTB minigene clones and the base pairing between the modified U1 snRNAs and the 5′ss of mutant exons. pTB exons and the exon of interests are represented by grey and colored boxes, respectively. Intronic sequences of pTB and flanking intronic regions of the exon of interest are represented by thin and thick lines, respectively. Black arrows stand for forward and reverse primers for RT-PCR amplification. (b, e) RT-PCR analysis of HeLa cells transfected with WT/mutant pTB minigenes and WT/modified U1 snRNAs. RT-PCR products were sequenced and schematically illustrated. (c, f) The histogram represents the densitometric analysis of the splicing products normalized to GAPDH (∗∗∗∗ represents *P* < 0.0001). Data are presented as the mean ± SEM (*n* = 3). M: marker; WT: wild type; MUT: mutant.

To demonstrate the effect of the c.2566+4A>G variant in intron 20 of the *DDB1* gene on pre-mRNA splicing, WT and MUT *DDB1* pTB minigenes covering exon 20 and flanking intronic sequences were transfected into HeLa cells. Transfection with WT *DDB1* minigene resulted in a correctly spliced 409 bp product, and transfection with MUT *DDB1* minigene resulted in a prevalent 244 bp product which represented the total exclusion of exon 20 together with residual correctly spliced 409 bp product (Figure 2(b)). While a decrease in the authentic 5′ss score (HSF 3.1 score from 94.1 to 86.91) was reported, the sequence analysis interface at HSF reported an increased splice site score (HSF 3.1 score from 75.44 to 85.53) for the cryptic 5′ss (GT dinucleotide at the position +5 and +6). Sanger sequencing showed the correct nature of the 409 bp product, suggesting that the cryptic 5′ss GT dinucleotide with weaker HSF 5′ss score did not prevent exon skipping.

To analyze the effect of the fourth variant c.1026+6T>A in the intron 7 of the *CRPPA* gene, HeLa cells were transfected with WT and MUT *CRPPA* pTB minigenes covering exon 7 and flanking intronic sequences. Transfection with WT *CRPPA* minigene resulted in a correctly spliced 337 bp product while transfection with MUT *CRPPA* minigene resulted in two products: the most favored correctly spliced 337 bp product and the 244 bp product representing the exon 7 exclusion (Figure 2(e)).

**Figure 3:**
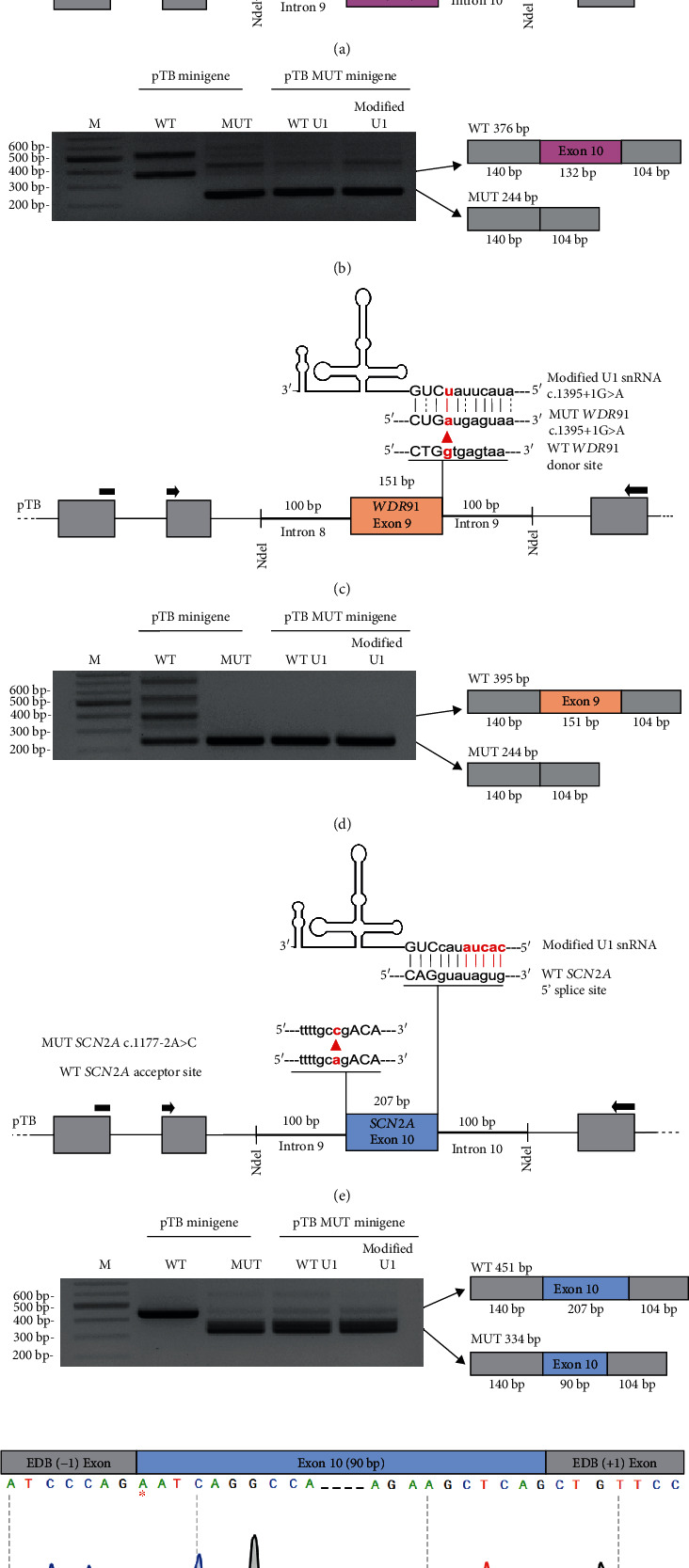
Characterization of *PLOD1* c.1116+2_1116+3insTT, *WDR91* c.1395+1G>A, and *SCN2A* c.1177-2A>C variants and application of modified U1 snRNAs for mis-splicing rescue. (a, c, e) Schematic representations of the location of variants on pTB minigene clones and the base pairing between the modified U1 snRNAs and the 5′ss of mutant exons. pTB exons and the exon of interests are represented by grey and colored boxes, respectively. Intronic sequences of pTB and flanking intronic regions of the exon of interest are represented by thin and thick lines, respectively. Black arrows stand for forward and reverse primers for RT-PCR amplification. (b, d, f) RT-PCR analysis of HeLa cells transfected with WT/mutant pTB minigenes and WT/modified U1 snRNAs. The asterisk indicates the first nucleotide (*SCN2A* (NM_001040142.2): c.1294) of the partially excluded *SCN2A* exon 10. RT-PCR products were sequenced and schematically illustrated. M: marker; WT: wild type; MUT: mutant.

The c.1116+2_1116+3insTT variant in intron 10 of the *PLOD1* gene was previously reported in a female patient with an unknown origin [37]. Although the effect of the variant on pre-mRNA splicing had already been shown in patient dermal fibroblasts, to further test the effect of U1 snRNA, WT and MUT *PLOD1* pTB minigenes covering exon 10 and flanking intronic sequences were first expressed in HeLa cells. Transfection with WT *PLOD1* minigene resulted in two products: a correctly spliced 376 bp product and an aberrant 536 bp product. Sanger sequencing showed that the aberrant 536 bp product included 60 bp from the 3′ end of the pTB-resident EDB-1 intron and 100 bp from the *PLOD1* intron 9 and correctly spliced 376 bp fragment. Transfection with a MUT *PLOD1* minigene resulted in the most abundant 244 bp product which is generated by the total exclusion of exon 10 and several unknown products (Figure 3(b)).

To demonstrate the effect of the c.1395+1G>A variant in the intron 9 of the *WDR91* gene on pre-mRNA splicing, WT and MUT *WDR91* pTB minigenes covering exon 9 and flanking intronic sequences were transfected into HeLa cells. Transfection with WT *WDR91* minigene resulted in four products: 244 bp, 395 bp, and aberrant ~420 bp and ~700 bp products. While the 395 bp product corresponds to the correct splicing of exon 9, the 244 bp product indicated the total exclusion of exon 9. Transfection with a MUT *WDR91* minigene resulted in only the 244 bp product being present, which corresponds to the exon 9-excluded transcript (Figure 3(d)).

Finally, to analyze the effect of the last identified variant c.1177-2A>C in the intron 9 of the *SCN2A* gene, HeLa cells were transfected with WT and MUT *SCN2A* pTB minigenes covering exon 10 and flanking intronic sequences. Transfection with WT *SCN2A* minigene resulted in a correctly spliced 451 bp product. Transfection with MUT *SCN2A* minigene resulted in three products: 334 bp, 451 bp, and an aberrant 611 bp products. Sanger sequencing showed that the most prevalent 334 bp product represents the partial (117 bp) exclusion of the 5′ end of exon 10 due to the activation of a cryptic exonic 3′ss (AG dinucleotide at the position just before the *SCN2A* (NM_001040142.2): c.1294) (Figure 3(f)). It is predicted that the variant does not cause any shift in the reading frame. Sequencing also showed that the aberrant 611 bp product includes 60 bp from the 3′ end of the pTB-resident EDB-1 intron, 100 bp from *SCN2A* intron 9, and the correctly spliced 451 bp transcript.

### 3.3. Modified U1 snRNAs Partially Rescued Four of the Selected Variants

Except for *SCN2A* c.1177-2A>C variant, all variants we analyzed for their effect on pre-mRNA splicing are at the 5′ splice site. To improve the reduced complementarity of the U1 snRNA tail to 5′ss, we generated modified U1 snRNA expressing plasmids by site-directed mutagenesis for each variant. For each case, MUT pTB plasmids that induced mis-splicing were cotransfected with WT and modified U1 snRNA vector into HeLa cells to test the potential therapeutic effect of U1 snRNAs containing the nucleotide changes of interest.

For the MUT *ERCC6*, *PTPMT1*, *DDB1*, and *CRPPA* minigenes, cotransfection with modified U1 snRNAs partially rescued the mis-splicing phenotype in HeLa cells.

Specifically, for the *ERCC6* c.1992+3A>G variant, a U1 snRNA carrying a single nucleotide modification (+3C) partially rescued splicing, as evidenced by the expression of correctly spliced 415 bp product. However, the rescue was not complete because a 333 bp product with the partial exon 9 exclusion was still present at a low level (Figures 1(b) and 1(c)).

For the *PTPMT1* c.255G>C variant, a U1 snRNA carrying a single nucleotide modification (-1G) partially rescued splicing, as evidenced by the appearance of correctly spliced 325 bp product and a reduction in the fraction of the exon 2-excluded 244 bp product. Nonetheless, a 487 bp product representing 162 bp of intron 2 retention was still present with a reduced percentage (Figure 1(e) and 1(f)).

For the *DDB1* c.2566+4A>G variant, U1 snRNA carrying a two-nucleotide modification (-2A, +4C) partially rescued splicing by inducing the expression of correctly spliced 409 bp product and reducing the 244 bp product which corresponds to the total exclusion of exon 20. As in the other cases, a low level of 244 bp product which represents the total exclusion of exon 20 was still detectable (Figures 2(b) and 2(c)).

Ultimately, for the *CRPPA* c.1026+6T>A variant, it appears that the U1 snRNA carrying a two-nucleotide modification (-1A, +6T) rescued the low-level mis-spliced transcript, promoting the expression of correctly spliced 337 bp product (Figures 2(e) and 2(f)).

Collectively, these findings imply that the use of modified U1 snRNAs for therapeutic purposes to correct aberrant splicing holds promise for achieving beneficial consequences, at least for some of the splicing variants.

## 4. Discussion

Around 6,000 to 8,000 rare diseases affect 8–10% of the worldwide population. It is estimated that rare diseases affect more than 6 million people in Türkiye, which has a high rate of consanguineous marriage (24%) [38].

Around 95% of human genes undergo alternative splicing. Variants that affect pre-mRNA splicing are one of the major causes of rare disease formation. About 15–30% of the variations thought to be responsible for genetic diseases have an impact on splicing by whole/partial exon skipping or whole/partial intron retention [39, 40].

In our recent study, we applied WES in a cohort of clinically undiagnosed 246 children from 190 consanguineous families. We identified 27 novel genes and 119 known disease genes with an 86% diagnostic rate overall. Among the identified variants, 15 of them (~10%) were novel or previously reported splicing variants [22].

Identified variants were first analyzed *in silico* with varSEAK and HSF 3.1 tools to predict the effects of mutations on pre-mRNA splicing. Then, variants included in the study were selected according to these criteria: splicing effect score and rescue potential with a modified U1 snRNA (mostly variants at 5′ss resulting in exon skipping). By considering these criteria, seven splicing variants were included in this study. Two of them (*ERCC6* c.1992+3A>G and *PLOD1* c.1116+2_1116+3insTT) were previously reported variants [36, 37] while the splicing impact of other five variants (*WDR91* c.1395+1G>A, *PTPMT1* c.255G>C, *SCN2A* c.1177-2A>C, *DDB1* c.2566+4A>G, and *CRPPA* c.1026+6T>A) was reported for the first time in our cohort.

Minigene-based assays are important strategies to evaluate the effect of a specific DNA variant on pre-mRNA splicing, especially in the absence of patient-derived samples [41]. In this study, we now show that all seven variants analyzed disrupted the splicing pattern. Furthermore, we then applied a modified U1 snRNA approach to correct variant-induced splice defects in HeLa cells. Interestingly, modified U1 snRNAs provided partial rescue for four of the selected variants, suggesting that, for some splicing variants, this could represent a potentially good therapeutic strategy to be further investigated.

To the best of our knowledge, there is no previous patient reported with a pathogenic variant in the *PTPMT1* gene. After the first patient included in this study was reported from Türkiye [22], two different patients presenting similar clinical findings with different national backgrounds, one carrying the same splicing variant and the other with a different missense variant in the *PTPMT1* gene, have been reported (currently under review). These subsequent findings provide additional evidence supporting the hypothesis that the *PTPMT1* gene is implicated in the development of the observed phenotype. Therefore, the effect of c.255G>C splice site variant in the *PTPMT1* gene encoding a mitochondrial phosphoinositide phosphatase was tested in the more natural context of a patient fibroblast-derived RNA sample. Sanger sequencing of the patient sample showed the recognition of the cryptic splice site at the 162nd nucleotide position in the 5′ end of intron 2 (Supplementary Figure S1). We have re-evaluated the splicing defect in HeLa cells to further test the U1 snRNA-mediated therapeutic correction of mis-splicing. Importantly, partial correction of the splicing defect caused by the c.255G>C variant could be achieved with a designed modified U1 snRNA (-1G) in HeLa cells (Figures 1(e) and 1(f)). Despite testing the same modified U1 snRNA in patient fibroblasts, the transfection efficiency was insufficient to evaluate its effects conclusively, thereby hindering the determination of the potential therapeutic effects of modified U1 snRNA in these cells. Further studies are needed in which this modified U1 snRNA is expressed at high levels in the patient fibroblasts, thereby allowing for a more conclusive assessment of its effectiveness in a more physiologically relevant context.

Different from others, to show the preferred cryptic splice site at nucleotide position 162 in intron 2, the *PTPMT1* minigene construct was generated by including 42 bp from intron 1 and 245 bp from intron 2 (not 100 bp from both flanking introns). The 100 bp of the 5′-flanking region of the intron 2 was not enough to catch all potential cryptic sites, reaffirming the requirement of approximately 200 bp of the 5′ and 3′ flanking intronic regions [42].

While the U1 snRNA engineered for *PTPMT1* c.255G>C (p.Glu85His) variant partially rescues the splicing defect, it is noteworthy that the presence of a missense variant at the 3′ terminus of the exon 2 persists within the resultant protein structure. Accordingly, another important concern is whether, even in the case of rescue, the missense variant within the protein might influence its stability and functionality, as the p.Glu85His variant resides in the phosphatase domain. The substitution replaces the polar uncharged glutamic acid with the positively charged histidine residue. We utilized a combination of web-based computational tools, including PremPS (https://lilab.jysw.suda.edu.cn/research/PremPS/) [43], I-Mutant 2.0 (https://folding.biofold.org/i-mutant/i-mutant2.0.html) [44], and DDMut (https://biosig.lab.uq.edu.au/ddmut/) [45], to analyze the effect of the p.Glu85His mutation on protein stability by assessing the Gibbs free energy change (ΔΔ*G*) between the wild-type and mutant forms of the protein. PremPS (ΔΔ*G* = 0.18 kcal/mol) and I-Mutant 2.0 (ΔΔ*G* = −0.77 kcal/mol) computational tools indicate destabilizing effects, whereas DDMut (ΔΔ*G* = 0.11 kcal/mol) suggests stabilizing effect for the variant. Although noncovalent interactions with neighboring residues, as identified by PremPS, exhibit no alteration between the wild-type and mutant forms, DDMut analysis indicates the formation of an additional hydrogen bond between the His85 and Ser83 residues in the mutant form. Furthermore, the Gln85 residue is located at the protein surface, and the protein structure of the mutant form predicted by the SWISS-MODEL (https://swissmodel.expasy.org/) [46] server suggests that the p.Gln85His substitution does not significantly affect the overall conformation of the protein. Therefore, after the partial splicing rescue, it is highly probable that the resultant PTPMT1 protein with p.Glu85His variant will be functional.

The c.1992+3A>G variant in the *ERCC6* gene encoding the DNA excision repair protein Cockayne syndrome B (CSB) was previously reported in the six members of another Turkish family with similar clinical findings [36]. We re-evaluated the mis-splicing in *ERCC6* pre-mRNA in HeLa cells and developed mutation-adapted U1 snRNA to rescue the defective splicing. Modified U1 snRNA with a single nucleotide modification (+3C) successfully increased the level of correctly spliced transcript (Figures 1(b) and 1(c)). If these two families do not have a common ancestor, we can here talk about the possible founder effect of the *ERCC6* c.1992+3A>G variant. Therefore, manipulation of *ERCC6* mis-splicing caused by a c.1992+3A>G variant with a modified U1 snRNA could be an important therapeutic strategy for the Turkish population.

Another case of partial rescue provided by the modified U1 snRNA approach is a female patient with a c.2566+4A>G variant in the *DDB1* gene. The five different *de novo* variants in the monofunctional DNA-alkylating methyl methanesulfonate (MMS1) domain of *DDB1* have been previously identified in eight unrelated pediatric patients primarily exhibiting symptoms such as hypotonia, developmental delay, cognitive impairment, obesity, skeletal abnormalities, and dysmorphic features (OMIM: 619426) with an autosomal dominant inheritance. Authors suggested dominant-negative or gain-of-function mechanisms for these variants [26]. In our cohort, unlike previous cases, a novel autosomal recessive inheritance pattern was identified for the *DDB1* gene, as it occurs in most other DNA repair disorders. Specifically, the c.2566+4A>G variant causes an in-frame deletion of 35 amino acids in the cleavage and polyadenylation specificity factor (CPSF) domain in the DDB1 protein. This variant was found to be homozygous in the patient while being heterozygous in her healthy parents [22]. Our patient, reported with a c.2566+4A>G variant in the biallelic state, exhibits clinical findings (Table 1) quite like those of previously reported patients with different variants in the monoallelic state. The coexistence of a portion of wild-type transcript alongside the exon 20 skipped transcript in HeLa cells transfected with mutant *DDB1* minigene could also be consistent with the dominant-negative or loss-of-function mechanisms for the c.2566+4A>G variant. Nevertheless, the observation of the monoallelic state of the c.2566+4A>G splicing variant in asymptomatic parents could be explained by the presence of a variant affecting a different domain than those reported in the literature, or by the fact that the splicing variant effect has a threshold keeping it below a critical level in the parents. For this reason, the impact of the c.2566+4A>G variant in the *DDB1* gene should also undergo testing in a more natural context, such as a patient-derived fibroblast RNA sample.

The patient with the homozygous c.1026+6T>G variant in the *CRPPA* gene is included in the congenital muscular dystrophy-dystroglycanopathy with brain and eye anomalies (type A) (OMIM: 614643) group due to clinical findings such as congenital hypotonia, delayed motor milestones, cognitive dysfunction, ophthalmoparesis, muscle weakness, and increased creatine kinase (Table 1), as reported in literature [27]. Multiexon deletions and splicing variant-induced exon skippings in the *CRPPA* gene are frequent, and homozygous [47], heterozygous [48], or compound heterozygous [47, 49] splicing variants have been reported. While the clinical findings of our patient show high compatibility with patients reported in the literature, a very low level of exon 7 skipped transcript was observed in HeLa cells transfected with MUT *CRPPA* minigene. A plausible explanation could be that the splicing outcome observed through the minigene approach in HeLa cells may differ from that of patient fibroblasts. Although it was not possible to demonstrate the splicing defect in the patient material for the case in question, the fact that we did not observe the correct transcript in patient dermal fibroblasts carrying *PTPMT1* c.255G>C variant, despite seeing it in the minigene assay, supports the notion that a similar discrepancy may be present for the *CRPPA* variant (Supplementary Figure S1 and Figure 1(e)), and further work will be required to clarify this issue. As it stands, the evidence we provide in this study is not conclusive enough to support a disease-causing effect for this variant.

The c.1116+2_1116+3insTT variant in the *PLOD1* gene encoding a lysyl hydroxylase 1 enzyme which converts lysine to a hydroxylysine through hydroxylation was also previously reported in a female patient of an unknown origin with an Ehlers-Danlos syndromes (EDS) type IV [37]. Here, the splicing effect of the c.1116+2_1116+3insTT variant detected in another female patient in our cohort with very similar clinical characteristics was re-evaluated. Application of modified U1 snRNA carrying a two-nucleotide modification (+3A, +4A) did not improve the recognition of mutated 5′ss in the intron 10 and correct splicing (Figure 3(b)).

Moreover, the *WDR91* gene encoding an endosomal protein implicated in early to late endosome conversion has not been linked to any pathological conditions before. After its initial identification within our cohort [22], another patient exhibiting very similar clinical findings (developmental delay, microcephaly, and seizures) and harboring another novel splicing variant (NM_014149.4: c.511+1A>G) was recently reported in an Italian pediatric cohort [50]. Together with in vivo functional studies demonstrating the effect of WDR91 in neuronal development [30, 31], the second reported patient with a novel splicing variant in the *WDR91* gene provided crucial evidence regarding its implication in disease etiology. The difficulty of rescuing splicing defects caused by variants affecting the invariant GT dinucleotide at 5′ss, as demonstrated in previous studies [51], was also observed for the *WDR91* (NM_014149.4): c.1395+1G>A variant. The application of modified U1 snRNA carrying a proper nucleotide modification (+1T) did not result in enhanced recognition of the mutated 5′ss within intron 9, nor did not rescue the aberrant splicing. Furthermore, for the two additional aberrant transcripts (~420 bp and ~700 bp) observed in HeLa cells transfected with WT *WDR91* minigene, it can be hypothesized that the failure to achieve sequencing for these two PCR products indicates potential heteroduplex formation.

All of the modified U1 snRNAs used in this study have one or two modified nucleotides. For the cases that did not show effective rescue with the use of the corresponding modified U1 snRNAs, U1 snRNAs with different degrees of complementarity to 5′ss could be used to improve the normal splicing process.

In addition to our findings, we should also mention some limitations of our approach. First of all, the maintenance of the genomic context, including intron length, is essential for exon recognition and, therefore, for minigene design [52–55]. Unfortunately, no patient RNA samples for many of the spliceogenic variants in the present study were available. However, it should also be considered that even the availability of patient RNA does not always represent an optimal solution, and limitations are also present for this material. For example, in many cases, the most readily available patient RNA comes from blood PBMCs that not always may reflect the original cellular context. Therefore, also in this case, researchers should consider the presence/absence of tissue-specific alternative splicing that may not be easy to replicate unless specific splicing regulators are knocked out or overexpressed, as recently shown when comparing the splicing profile of a well-known splicing factor, TDP-43, in neuronal vs. muscle cell lines [56]. In addition, results from patient RNA may also be difficult to interpret due to the expression of the wild-type allele that may hide the production of the full-length transcript, thus resulting in incorrect clinical interpretation of a specific variant. Nevertheless, regarding this issue, in this work, we provide evidence that at least in the case of one mutation, the splicing observed in fibroblasts can mimic the results of the minigene. In conclusion, therefore, although there is still no perfect method to check variant-splicing outcomes, our minigene assays provide a cost-effective and rapid method to characterize both variant-induced transcripts in a set of diverse genes and it also represents a rapid way to test for potential rescue strategies (such as the modified U1 snRNA approach used in our study).

For the in vivo delivery of engineered U1 snRNAs as RNA therapeutics, adeno-associated virus (AAV) vectors have been the most preferred delivery method [57–60]. To date, in various clinical trials, AAV-based gene therapies have been used mainly to target the central nervous system (CNS), liver, and muscles via intravenous or local injections [61]. Moreover, the translation of the efficacious modified U1 snRNAs designed in this work for the splicing variants in *ERCC6*, *PTPMT1*, *DDB1*, and *CRPPA* genes to the clinic could also be possible through AAV-mediated delivery specifically targeting the neuromuscular system components. While the functions of these genes may vary, they are predominantly expressed within tissues such as the brain, muscle, and endocrine tissue. Consequently, patients develop disorders that commonly manifest in brain and muscle development. In contrast to prior therapeutic constraints, AAV-based gene therapy is a promising therapeutic approach with high tissue specificity, long-term transgene expression, high delivery efficiency, reduced immunogenicity, and pathogenicity with improved AAV serotypes for diverse human diseases today [18, 62].

Overall, the results of our study show that the in vitro minigene assay can provide useful information on the splicing effect of five novel splicing variants identified in a cohort of Turkish pediatric patients with rare neurogenetic conditions. Additionally, the effects of two previously reported variants on splicing alteration were re-evaluated, and we provided evidence for the potential reclassification of some of the studied variants that were previously classified as likely pathogenic or VUS according to ACMG guidelines.

Finally, by partially rescuing mis-splicing with a modified U1 snRNA in four of the studied cases, we have demonstrated the potential applicability of the U1 snRNA-mediated therapeutic approach for a group of patients with different rare neurogenetic conditions, under in vitro conditions. However, translating this approach into clinical practice still requires further in vitro and in vivo studies to thoroughly evaluate its efficacy and potential side effects. Nonetheless, in populations with a high consanguinity rate, the correct classification of rare disease variants and the potential use of therapeutic strategies based on U1 snRNA can help improve patient management and reduce the economic burden of rare diseases.

## Figures and Tables

**Table 1:** Summary of patient data and major clinical findings.

Sample name	Age	Sex	Origin	Gene	Variant^a^	ACMG classification^b^	Major clinical findings
FMAL041	14 y.o.	M	Turkish	*ERCC6*	(NM_000124.4): c.1992+3A>G	Likely pathogenic	Psychomotor retardation, cognitive impairment, conductive hearing impairment, tremor of the hands, dysarthria, and dysmorphic findings (flat forehead, thick eyebrows, deltoid hypertrophy)
FIZM011	11 y.o.	M	Turkish	*PTPMT1*	(NM_175732.3): c.255G>C p.Gln85His	Uncertain significance	Psychomotor retardation, cognitive impairment, cerebral and cerebellar atrophy, bulbar signs, ophthalmoparesis, nystagmus, and sensorineural hearing impairment
FMAL057	5 y.o.	F	Turkish	*DDB1*	(NM_001923.5): c.2566+4A>G	Uncertain significance	Motor retardation, delayed speech, bulbar signs, spasticity, flexion contractures in upper and lower extremities, difficulty in walking, syndactyly, dysmorphic findings, and lactic acidosis
FMAL027	2 y.o.	F	Turkish	*CRPPA*	(NM_001101426.4): c.1026+6T>A	Uncertain significance	Delayed motor milestones, cognitive impairment, ophthalmoparesis, muscle weakness, elevated creatine kinase
FMAL083	1 y.o.	F	Turkish	*PLOD1*	(NM_001316320.2): c.1116+2_1116+3insTT	Likely pathogenic	Motor retardation, delayed speech, hypotonia, congenital hip dislocation, muscle weakness, skeletal muscle atrophy, scoliosis, difficulty in walking
FMAL006	3 y.o.	F	Turkish	*WDR91*	(NM_014149.4): c.1395+1G>A	Likely pathogenic	Psychomotor retardation, cognitive impairment, seizures, spasticity, dystonia, chorea, ophthalmoparesis, bulbar signs, skeletal muscle atrophy, microcephaly, scoliosis, hypertrichosis
FMAL084	12 y.o.	M	Turkish	*SCN2A*	(NM_001040142.2): c.1177-2A>C	Pathogenic	Psychomotor retardation, cognitive impairment, seizures, scoliosis, dystonia, muscle weakness, inability to walk

^a^According to mapping by the Genome Reference Consortium Human Build 37 (GRCh37)/hg19. ^b^ACMG-The American College of Medical Genetics and Genomics, by VarSome germline variant classifier (https://varsome.com).

## Data Availability

All data used to support the findings of this study are included within the manuscript's main text or supporting information. Previously reported variant data supporting this study are available at ClinVar or LOVD databases (https://databases.lovd.nl/shared/individuals?search_created_by=04602, ClinVar variation IDs #1459856, #2500940 and #2500963).

## Supplementary Materials

Supplementary 1 Figure S1: alternative splicing of PTPMT1 pre-mRNAs in patient dermal fibroblasts harboring c.255G>C variant.

Supplementary 2 Table S1: list of the oligonucleotide sequences for PCR amplifications in this study.
