# Supplementary material for: Characterization and Engineered U1 snRNA Rescue of Splicing Variants in a Turkish Neurodevelopmental Disease Cohort
Source: Hum Mutat. 2024 May 28;2024:7760556. doi: 10.1155/2024/7760556 (PMC11925005; doi:10.1155/2024/7760556)

**Supplementary Figure S1.** Alternative splicing of *PTPMT1* pre-mRNA in patient dermal fibroblasts harboring c.255G>C variant. **(A)** Schematic representations of the exons of *PTPMT1* gene and location of c.255G>C (p.Gln85His) variant. Exons are represented by grey boxes and black arrows stand for forward and reverse primers for RT-PCR amplification. **(B)** Agarose gel electrophoresis of RT-PCR products amplified using primers flanking *PTPMT1* exon 2 and 3. **(C)** Sanger sequencing of RT-PCR products depicted in electropherograms. **(D)** Western blot analysis of PTPMT1 in patient and control fibroblasts. C: control, P1: patient 1, P2: patient 2


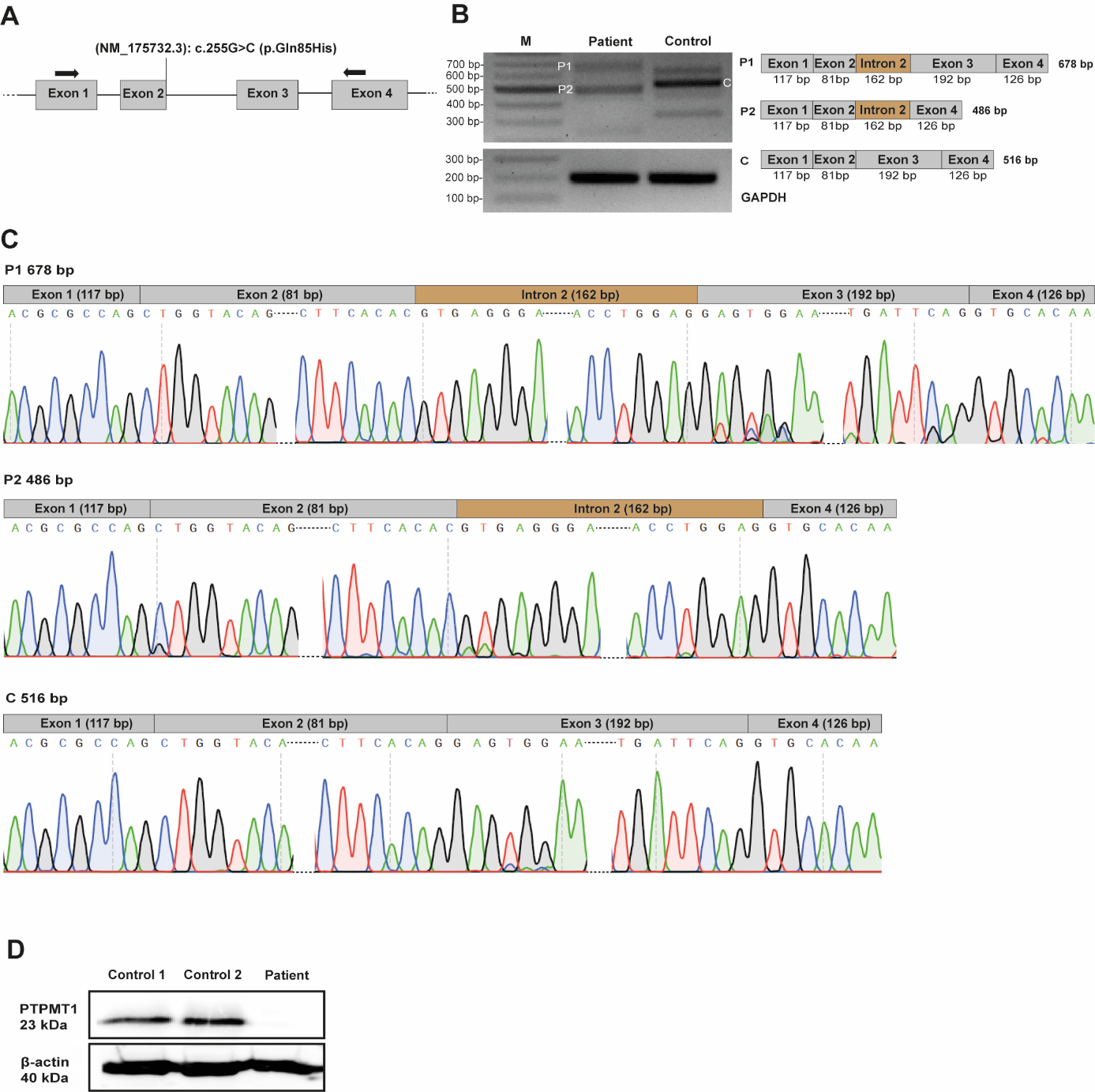

Supplement: Supplementary 1 — Figure S1: alternative splicing of PTPMT1 pre-mRNAs in patient dermal fibroblasts harboring c.255G>C variant. [file 7760556.f1.docx]
