# Supplementary material for: Characterization and Engineered U1 snRNA Rescue of Splicing Variants in a Turkish Neurodevelopmental Disease Cohort
Source: Hum Mutat. 2024 May 28;2024:7760556. doi: 10.1155/2024/7760556 (PMC11925005; doi:10.1155/2024/7760556)
Supplement: Supplementary 2 — Table S1: list of the oligonucleotide sequences for PCR amplifications in this study. [file 7760556.f2.docx]

**Supporting Information**

**Supplementary Table 1** List of the oligonucleotide sequences for PCR amplifications in this study

| **Name of oligonucleotide** | **Sequence (5’ to 3’)** |
| --- | --- |
| **Primers for *PTPMT1* Exon 2 cloning** |  |
| *PTPMT1* Exon 2 **F** | GGAATTCCATATGGGGTCTCCACCGTCTTTGCT |
| *PTPMT1* Exon 2 **R** | GGAATTCCATATGTGCTGGGATTACAGGCGTGA |
| **Primers for patient *PTPMT1* cDNA** |  |
| *PTPMT1* cDNA Exon 1 **F** | CTGCTCTACACCCTGTTCCG |
| *PTPMT1* cDNA Exon 4 **R** | TGTTGCCCGTGCAGTAATCT |
| **Standard pTB minigene primers** |  |
| Alfa 2‐3 **F** | CAACTTCAAGCTCCTAAGCCACTGC |
| BRA 2 **R** | GGTCACCAGGAAGTTGGTTAAATCA |
| **Primers for *GAPDH*** |  |
| *GAPDH* **F** | GGAGCGAGATCCCTCCAAAAT |
| *GAPDH* **R** | GGCTGTTGTCATACTTCTCATGG |
| **Primers for U1 snRNA site-directed mutagenesis** |  |
| *ERCC6* U1 c.1992+3A>G **F** | agatctcATACTCACCTGgcaggggaga |
| *ERCC6* U1 c.1992+3A>G **R** | tctcccctgcCAGGTGAGTATgagatct |
| *PTPMT1* U1 c.255G>C **F** | agatctcATACTTACGTGgcaggggaga |
| *PTPMT1* U1 c.255G>C **R** | tctcccctgcCACGTAAGTATgagatct |
| *DDB1* U1 c.2566+4A>G **F** | agatctcATACCTACCAGgcaggggaga |
| *DDB1* U1 c.2566+4A>G **R** | tctcccctgcCTGGTAGGTATgagatct |
| *CRPPA* U1 c.1026+6T>A **F** | agatctcATTCTTACATGgcaggggaga |
| *CRPPA* U1 c.1026+6T>A **R** | tctcccctgcCATGTAAGAATgagatct |
| *PLOD1* U1 c.1116+2_1116+3insTT **F** | agatctcATACAAACCTGgcaggggaga |
| *PLOD1* U1 c.1116+2_1116+3insTT **R** | tctcccctgcCAGGTTTGTATgagatct |
| *WDR91* U1 c.1395+1G>A **F** | agatctcATACTTATCTGgcaggggaga |
| *WDR91* U1 c.1395+1G>A **R** | tctcccctgcCAGATAAGTATgagatct |
| *SCN2A* U1 c.1177-2A>C **F** | agatctcCACTATACCTGgcaggggaga |
| *SCN2A* U1 c.1177-2A>C **R** | tctcccctgcCAGGTATAGTGgagatct |

**F**-Forward **R**-Reverse
